# CD70 drives cSCC growth by linking DNA damage response, inflammation, and tumor–stromal signaling

**DOI:** 10.21203/rs.3.rs-8206807/v1

**Published:** 2025-12-12

**Authors:** Tianshun Zhang, Qiushi Wang, Asad Khan, Chengcheng Hu, Emanuel Petricoin, Rebecca Morris, Sally Dickinson, Georg Wondrak, Ann.M Bode, Clara Curiel-Lewandrowski

**Affiliations:** The Hormel Institute, University of Minnesota; The Hormel Institute, University of Minnesota; The Hormel Institute, University of Minnesota; 3Department of Epidemiology and Biostatistics; Geroge Mason University; The Hormel Institute, University of Minnesota; The University of Arizona Cancer Center; University of Arizona; University of Minnesota; The University of Arizona Cancer Center

**Keywords:** CD70, non-melanoma skin cancer, UV-induced DNA damage, Inflammatory signaling, E2F1, keratinocytes, fibroblasts

## Abstract

Chronic ultraviolet (UV) exposure drives the development of non-melanoma skin cancers (NMSCs), particularly cutaneous squamous cell carcinoma (cSCC), through persistent DNA damage and inflammation. However, the molecular mediators that link genotoxic stress to tumor-promoting signaling and stromal activation remain poorly defined. Here, we identify CD70, a TNF superfamily member, as a UV- and DNA damage–inducible regulator that coordinates epithelial and stromal responses to promote skin carcinogenesis. Integrative analyses of transcriptomic (GTEx, GSE2503, GSE42677), proteomic (RPPA), and immunostaining datasets revealed marked upregulation of CD70 in sun-exposed skin, actinic keratoses, and cSCC lesions. Functionally, CD70 silencing suppressed cSCC proliferation and xenograft growth, whereas solar UV or DMBA exposure induced CD70 expression. Mechanistically, E2F1 directly bound and activated the CD70 promoter, establishing a transcriptional axis linking the DNA damage response to CD70 upregulation. CD70 depletion disrupted cytokine–receptor and MAPK/NF-κB signaling and altered inflammatory gene expression in UV-irradiated keratinocytes. In dermal fibroblasts, TGF-β–induced CD70 enhanced NF-κB activation and secretion of IL-6 and MCP3, thereby reinforcing paracrine inflammatory loops that supported cSCC spheroid expansion and tumor progression. CD70 knockdown in fibroblasts abrogated these effects and reduced tumor proliferation and cytokine expression in vivo. Collectively, our findings identify CD70 as a stress-inducible signaling hub that links DNA damage, inflammation, and tumor–stromal communication in skin carcinogenesis. Targeting CD70 may disrupt this feed-forward inflammatory circuit and provide a therapeutic strategy for inflammation-driven skin cancer.

## Introduction

Non-melanoma skin cancers (NMSCs), including actinic keratosis (AK) and cutaneous squamous cell carcinoma (cSCC), are the most common human malignancies, with rising incidence due to cumulative ultraviolet (UV) exposure and aging (1, 2). Chronic UV irradiation induces persistent DNA damage, oxidative stress, and inflammation, which are hallmarks of skin carcinogenesis (3, 4). Although mutations in oncogenic drivers such as *TP53, RAS*, and *NOTCH* have been well characterized (5–9), the molecular regulators that integrate DNA damage signaling, inflammation, and tumor–stroma interactions in NMSC remain poorly understood.

CD70, a type II transmembrane protein of the tumor necrosis factor (TNF) superfamily, is classically known for its role in adaptive immunity through CD27-mediated T-cell activation, proliferation, and differentiation (10–13). Aberrant CD70 expression has been reported in hematologic and solid malignancies, where it can contribute to immune evasion and tumor progression (10–13). Emerging evidence also indicates that CD70 can transmit tumor-intrinsic signals, engaging pathways such as PI3K/AKT and MAPK to promote proliferation, invasion, and epithelial–mesenchymal transition (10, 14–16). In these contexts, CD70 expression can be rapidly induced by inflammatory cytokines (12, 17), viral infections (18, 19), hypoxia (10, 20, 21), or genotoxic stress (22), despite its minimal expression in normal resting tissues (13). This inducible behavior suggests that CD70 may act as a stress-responsive integrator linking environmental insults to oncogenic, inflammatory, and stromal signaling. However, its mechanistic role in UV-driven epithelial carcinogenesis remains largely unexplored.

Tumor progression is increasingly recognized as a product of interactions between malignant epithelial cells and the tumor microenvironment (TME) (23–25). Cancer-associated fibroblasts (CAFs), through secretion of cytokines, chemokines, and growth factors, can reinforce malignant signaling and promote tumor initiation, progression, and therapy resistance (26–29). Whether CD70 expression extends beyond epithelial cells to modulate fibroblast activation and tumor–stroma crosstalk in UV-induced skin cancer is unknown.

In this study, we identify CD70 as a UV-inducible, DNA damage–responsive molecule upregulated in sun-exposed skin, AK, and cSCC. Functional analyses demonstrate that CD70 promotes keratinocyte and fibroblast proliferation, activates MAPK and NF-κB signaling, and enhances pro-inflammatory cytokine expression. Mechanistically, CD70 is directly regulated by the DNA damage–associated transcription factor E2F1. *In vivo* and *in vitro* models reveal that fibroblast CD70 supports tumor growth and establishes a pro-tumorigenic TME. We hypothesize that UV-induced DNA damage activates CD70 via E2F1, linking genotoxic stress with pro-inflammatory and stromal remodeling pathways in NMSC.

## Materials and Methods

### Transcriptomic data collection and analysis

RNA-seq data from sun-exposed and sun-protected human skin were obtained from the GTEx database, and GEO datasets (GSE2503, GSE42677) were merged and normalized using the *inSilicoMerging* and *limma* packages in R (v4.3.3). Differentially expressed genes (DEGs) were identified using empirical Bayes moderation with FDR < 0.05 and |log_2_FC| ≥ 1. RNA-seq profiling of HaCaT cells 24 h post solar-simulated light (SSL; 60 kJ/m^2^ UVA, 2.9 kJ/m^2^ UVB) exposure was performed, and DEGs were analyzed using the same pipeline. All analyses and visualizations were performed in R (v4.3.3) by using the tidyverse (v2.0.0), ggplot2 (v3.5.1), ggpubr (v0.6.0), pheatmap, dplyr (v1.1.4), and limma (v3.58.1) packages. All bioinformatic details are provided in **Supplementary Materials and Methods.**

#### Reverse phase microarray analysis (RPPA)

RPPA was performed as described previously (30–32). Cell lysates were printed on nitrocellulose slides, probed with validated antibodies, and visualized using a tyramide-based amplification system. Data were quantified using MicroVigene software, normalized to total protein, and expressed as net signal intensity.

#### Plasmid construction, cell culture, transfection, and lentiviral infection

Human E2F1, the pGL3 promoter vector, and packaging vectors (pMD2.0G and psPAX) were obtained from Addgene (Watertown, MA) The human CD70-pGL3 promoter vector was purchased from Thermo Fisher Scientific (Waltham, MA). Site-directed mutagenesis was performed using the QuikChange Site-Directed Mutagenesis Kit (Cat. No. 200518–5; Agilent Technologies, anta Clara, CA) with primers for the human CD70 promoter: Forward: 5’-TGCCCAGGCTGGATGCGCTGCTGCCGCACAGCTCACAGCAGC-3’ Reverse: 5’-GCTGCTGTGAGCTGTGCGGCAGCAGCGCATCCAGCCTGGGCA-3’ Lentiviral plasmids shCD70 (#1, TRCN0000007840; #2, TRCN0000007841) were purchased from the University of Minnesota Genomics Center (Minneapolis, MN). ShE2F1 constructs (VB900040–4032wtd, VB900040–4039muj) were obtained from Vector Builder Inc. (Chicago, IL). The pLKO.1-puro Non-Target shRNA Control Plasmid (shCon) was purchased from Sigma-Aldrich (Burlington, MA). All constructs were verified by restriction enzyme mapping, DNA sequencing, and BLAST analysis.

HaCaT and HEK 293T cells were from ATCC (Manassas, VA); A431 and SCC-12 cells from Thermo Fisher Scientific (Waltham, MA); NHDF from Lonza (Walkersville, MD). Cells were used within 10 passages. HaCaT and A431 were cultured in DMEM with 10% FBS and 1% antibiotics; SCC-12 in RPMI 1640 with L-glutamine, 10% FBS, 1% antibiotics, and 1% MEM non-essential amino acids; NHDF in DMEM with 1X MEM, 10% FBS, and 1% antibiotics. CM was collected from serum-starved cells (48 h) and stored at −80°C. NHDF were treated with TGF-β (PeproTech, Rocky Hill, NJ) for 2 weeks to generate NHDF-TGF-β.

Transient transfection was performed using iMFectin DNA Transfection Reagent (GenDEPOT, Katy, TX) at 60–70% confluence for 36–48 h. For stable knockdown, lentiviral plasmids (shCD70, shE2F1, or shCon) with packaging vectors were transfected into HEK 293T cells using iMFectin Poly DNA Transfection Reagent (GenDEPOT). Viral supernatants were collected at 48 h, filtered (0.45 μm; MilliporeSigma, Burlington, MA), and used to infect target cells with 8 μg/mL polybrene (Sigma-Aldrich). Infected cells were selected with puromycin (1.2 μg/mL; Sigma-Aldrich) for 48 h.

Mutated CD70 promoter constructs were generated using the QuickChange Lightning Site-Directed Mutagenesis Kit (Agilent Technologies, Santa Clara, CA) and confirmed by Genewiz (South Plainfield, NJ).

#### Western blot analysis

Western blotting was performed following previously established protocols(33). Primary antibodies were diluted 1:1000 and incubated overnight at 4°C, followed by incubation with HRP-conjugated secondary antibodies at a 1:5000 dilution. Protein bands were visualized using a chemiluminescent substrate (GE Healthcare Biosciences, Piscataway, NJ). Detailed information is provided in **Supplementary Materials and Methods.**

#### Spheroid 3D cell culture

Vitrogel Hydrogel Matrix (VHM01, TheWell Bioscience, North Brunswick, NJ) was used for spheroid 3D cell culture according to the manufacturer’s instructions. A431 and SCC12 cells (shCon or shCD70) were suspended in culture medium, and 1 mL of Vitrogel was mixed with 500 μL of the cell suspension. A total of 300 μL of the hydrogel–cell mixture was dispensed into a 24-well plate at a density of 1 × 10^5^ cells per well and allowed to gel at room temperature for 15 min. After gelation, 300 μL of medium were gently added to cover the hydrogel. Cultures were maintained at 37°C with medium changes every 48 h. After 10 days, cells were harvested for immunofluorescence analysis.

For co-culture experiments, NHDF (shCon) or NHDF-TGF-β (shCon or shCD70) were combined with A431 or SCC12 cells and processed using the same Vitrogel-based procedure described above.

### Chromatin immunoprecipitation (ChIP) and qPCR

ChIP was performed using the Magna ChIP A/G kit (MilliporeSigma, Burlington, MA, MAGNA0017) following the manufacturer’s instructions with minor optimizations. A431 cells (1 × 10^7^ per IP) were cross-linked in 18.5% formaldehyde for 10 min and quenched with 10X glycine for 5 min. Cells were washed with ice-cold PBS, lysed sequentially with Cell Lysis Buffer and Nuclear Lysis Buffer supplemented with protease inhibitor, and chromatin was sheared by sonication into ~ 200–1000 bp fragments.

Equal amounts of chromatin were incubated with ChIP-grade antibody (E2F1) or rabbit IgG (1 μg per IP) and captured with Protein A/G magnetic beads overnight at 4°C. Beads were washed with Low Salt, High Salt, LiCl, and TE buffers. Chromatin was eluted with ChIP Elution Buffer containing Proteinase K, and cross-links were reversed at 62°C for 2 h followed by 95°C for 10 min. DNA was purified using the kit spin columns and eluted in 50 μL.

Enrichment at target loci was quantified by qPCR using primers (F: AGTAACACCCTATCCTCTACCCC; R: GCGCCCGGCCTAAATTAATATT) R: GTGCAGTTCAGTGATCGTACAGG). Reactions contained 2 μL DNA, 12.5 μL SYBR Green master mix, and 1 μL primer mix. Cycling: 94°C for 10 min; 50 cycles of 94°C for 20 s and 60°C for 1 min. Data are presented as fold enrichment over IgG control.

#### Xenograft mouse models

All animal studies were conducted in accordance with protocols approved by the University of Minnesota Institutional Animal Care and Use Committee (IACUC, Minneapolis, MN; Protocol ID 2404–42009A). Xenograft experiments were performed to evaluate the effects of CD70 knockdown in A431 or NHDF cells.

For the first experiment, six-week-old athymic nude mice (Charles River Laboratories, Wilmington, MA) were subcutaneously injected in the right flank with 4 × 10^6^ A431 cells expressing either shCon or shCD70 (n = 6 per group).

In the second experiment, a mixture of 4 × 10^6^ A431 cells with 1.67 × 10^6^ NHDF or NHDF-TGF-β cells, expressing either shCon or shCD70, resuspended in 150 μL of a 1:1 PBS:Matrigel solution and injected subcutaneously into nude mice (n = 6 per group). This design allowed comparison of tumors formed by A431 cells alone, A431 cells co-injected with NHDF-TGF-β cells expressing shCon, and A431 cells co-injected with NHDF-TGF-β cells expressing shCD70. Tumor size and body weight were measured weekly. Tumor volume was calculated using the formula: tumor volume (mm^³^) = length × width^2^ × 0.52.

The detailed information of reagents and antibodies, RNA extraction (PureLink^™^ RNA Mini Kit, Invitrogen, Carlsbad, CA), sequencing library, and bioinformatic analysis, Western blot analysis, cell viability (MTT) assay, immunofluorescence analysis, real-time PCR, Luciferase Assay and Human cytokine antibody array are described in **Supplementary Materials and Methods.**

### Statistical analysis

Quantitative data are presented as means ± standard deviation (SD) or standard error (SE), based on at least 3 independent experiments or samples. Differences in gene expression among groups from GEO or GTEx datasets were assessed using the Wilcoxon rank-sum test. For RPPA data, Logarithmic transformation with base 2 (log2) was applied to expression levels of the RPPA analytes to achieve approximate normality. Box-and-whisker plots and waterfall plots were used to visually contrast distribution of expression levels across different groups. Expression levels were compared between groups by using generalized estimating equations (GEE), which adjusted for within-patient correlation (multiple patients contributed both sun protected and sun damaged skin samples to this study). The analysis was conducted using R (version 4.3.1). For experimental data, statistical significance was evaluated using either a Student’s *t*-test or one-way ANOVA, followed by post hoc multiple comparisons with Tukey’s HSD tests as appropriate. All analyses were conducted using SPSS software (version 23, IBM Corp., Armonk, NY) or R (v4.3.3). A *p*-value < 0.05 was considered statistically significant.

## Results

### CD70 is highly expressed in sun exposed skin and non-melanoma skin cancers

**Figure 1:**
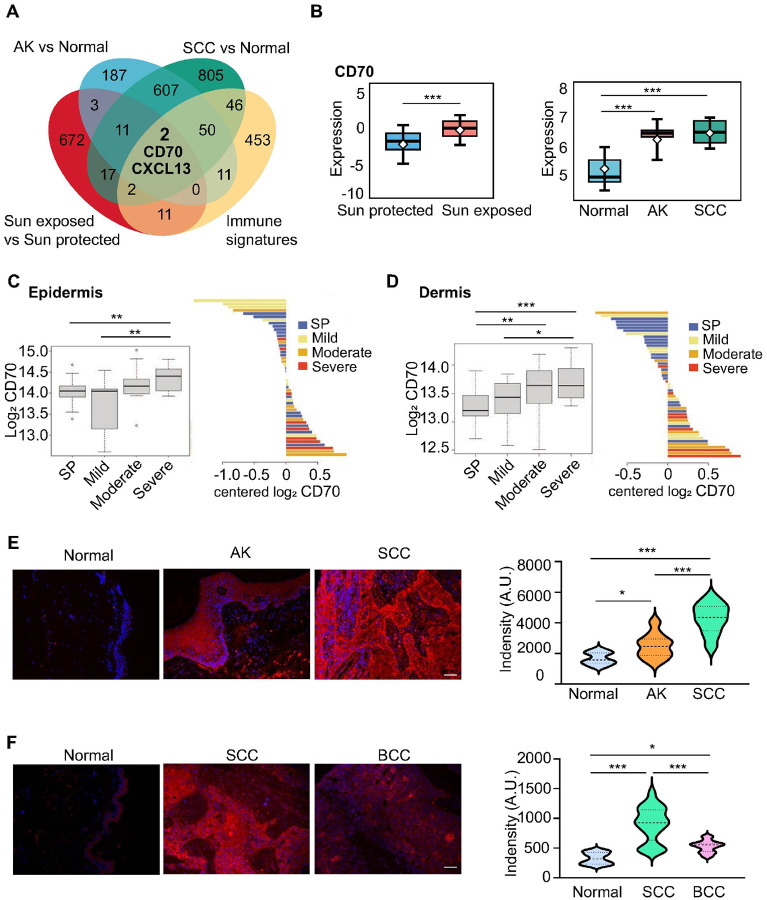
CD70 is upregulated in sun exposed skin and non-melanoma skin cancers. **(A)** Transcriptomic analysis of GTEx (sun exposed vs. sun protected skin) and GEO datasets (GSE2503, GSE42677; normal skin n=16, actinic keratosis [AK n=9], and cutaneous squamous cell carcinoma [cSCC n=15]) identified a variety of differentially expressed immune-related genes (FDR < 0.05, ≥2-fold). CD70 and CXCL13 were highlighted as candidates associated with sun exposure, immune response, and skin carcinogenesis. **(B)** CD70 expression is significantly higher in sun exposed skin compared with sun protected skin (left panel) and markedly elevated in AK and cSCC compared with normal controls (right panel). Statistical analysis was performed usingWilcoxon rank-sum test (***p< 0.001). Reverse-phase protein array (RPPA) analysis confirmed elevated CD70 expression in both epidermis **(C)** and dermis **(D)** of sun-damaged skin. Skin samples included 21 from sun-protected (SP) areas and 28 from sun-damaged (SD) areas, comprising 10 mild, 10 moderate, and 8 severe cases. **(E)** Immunofluorescence staining of human skin sections shows increased CD70 expression in AK (n=10) and cSCC (n=10) compared to normal skin (n=10). **(F)**immunofluorescence further revealed significant CD70 induction in both SCC (n=43) and BCC (n=13) compared to normal skin (n=10), with the strongest upregulation observed in SCC. CD70 is shown in red, and nuclei are counterstained with DAPI (blue). Scale bar: 50 μm. Statistical significance was determined by one-way ANOVA (Tukey’s HSD) vs control (SP or Normal; *, *p* < 0.05; **, *p* < 0.01; ***, *p* < 0.001).

To identify immune genes dysregulated in UV-exposed skin and NMSC, we analyzed GTEx transcriptomic data (sun exposed vs. sun protected skin) and merged GEO datasets GSE2503 and GSE42677 (AK, cSCC, and normal skin). Significant genes (FDR < 0.05, ≥ 2-fold change) overlapping with immune signature genes (34, 35) identified CD70 and CXCL13 as linked to sun exposure, immune responses, and skin carcinogenesis (Fig. 1A). CD70 was significantly upregulated in sun-exposed skin versus protected sites and markedly elevated in AK and cSCC compared with normal skin (Fig. 1B). CXCL13 was reduced in sun-exposed skin but increased in AK and cSCC (**Supplementary Fig. 1**). Based on these results, we focused on CD70 for mechanistic studies in UV-induced skin damage and carcinogenesis.

Consistently, Reverse-phase protein array (RPPA) showed higher CD70 expression in both epidermis (Fig. 1C) and dermis (Fig. 1D) of sun-damaged skin (mild to severe) versus protected skin. Immunofluorescence of human skin confirmed CD70 upregulation in AK and cSCC specimens (Fig. 1E; University of Arizona, HSC #03–65; IRB #11–0212-04, #12–0229-04). Immunofluorescence also showed elevated CD70 in SCC and BCC, with the strongest induction in SCC (SK801c, TissueArray.Com LLC, Derwood, MD) (Fig. 1F). These findings indicate that CD70 is a UV-inducible gene activated early in skin carcinogenesis and maintained through disease progression.

#### CD70 promotes growth of cSCC cells in vitro and in vivo

**Figure 2:**
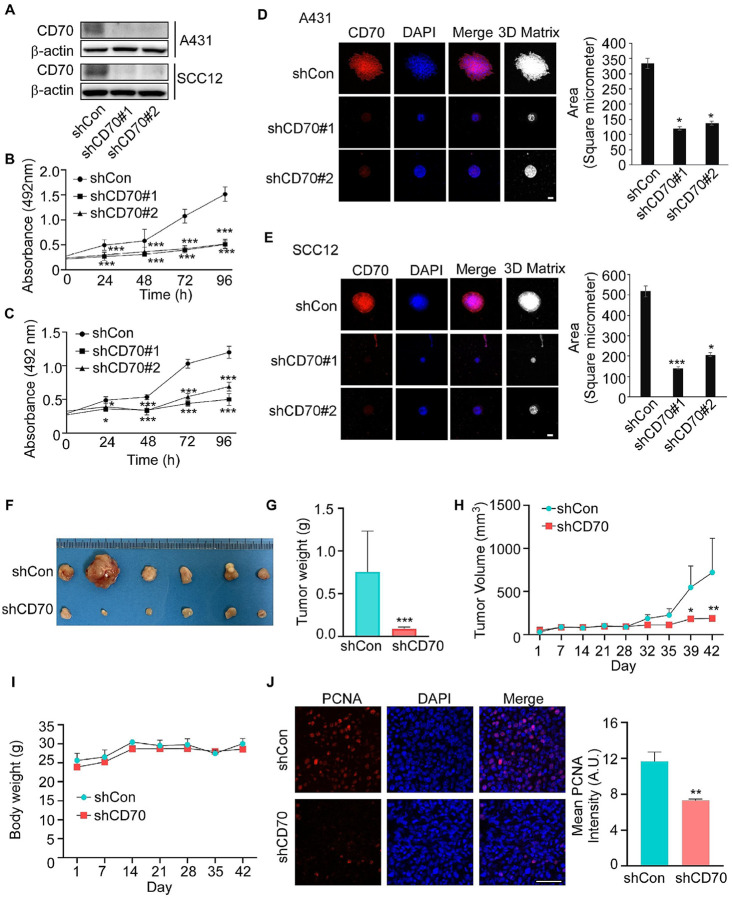
Knockdown of CD70 suppresses cSCC cell growth *in vitro* and in a xenograft mouse model. **(A)** A431 and SCC12 cells with stable shCon or shCD70 knockdown were established, and CD70 expression was confirmed by Western blotting. CD70 knockdown reduces the proliferation of **(B)** A431 and **(C)** SCC12 cells as measured by MTS assays.**(D, E)** CD70 knockdown decreases spheroid growth of A431 and SCC12 cells in 3D spheroid assays. CD70 is shown in red, and nuclei are counterstained with DAPI (blue). Scale bar: 50 μm. *In vivo* xenograft assays using A431 cells with stable CD70 knockdown demonstrate reduced tumor **(F)** size,**(G)** weight, and **(H)** volume compared with controls. (I) Body weight measurements show no significant differences between groups.(J)Immunofluorescence staining reveal decreased PCNA expression in CD70-silenced tumors (left), which was quantified (right) using the ZEISS ZEN 3.7 software program. Asterisks indicate statistical significance (*p < 0.05; **p < 0.01; ***p < 0.001 vs. shCon in each time point; Student’s *t*-test).

To evaluate the functional significance of CD70 in skin cancer cells, we knocked down CD70 expression by using specific shRNAs in human cSCC cell lines (A431 and SCC12). Knockdown efficiency in A431 and SCC12 cells was confirmed by Western blotting (Fig. 2A). Knockdown of CD70 significantly reduced cell proliferation, as measured by MTS assays (Fig. 2B, C); and suppressed spheroid formation in a 3D culture system (Fig. 2D, E), indicating impaired tumorigenic capacity.

To extend these findings *in vivo*, A431 cells stably expressing CD70 shRNA or scrambled control were subcutaneously implanted into immunodeficient nude mice. Tumors derived from CD70-silenced cells exhibited significantly reduced tumor size (**representative images**, Fig. 2F), weight (Fig. 2G), and volume (Fig. 2H) compared with controls but had no significant overall effect on body weight (Fig. 2I). Histological analysis (IF) further revealed decreased proliferation in CD70 knockdown tumors, as evidenced by reduced proliferating cell nuclear antigen (PCNA) staining (Fig. 2J). Together, these results support a pro-proliferative role for CD70 in cSCC progression.

### CD70 is UV- and DNA damage–inducible in keratinocytes

**Figure 3:**
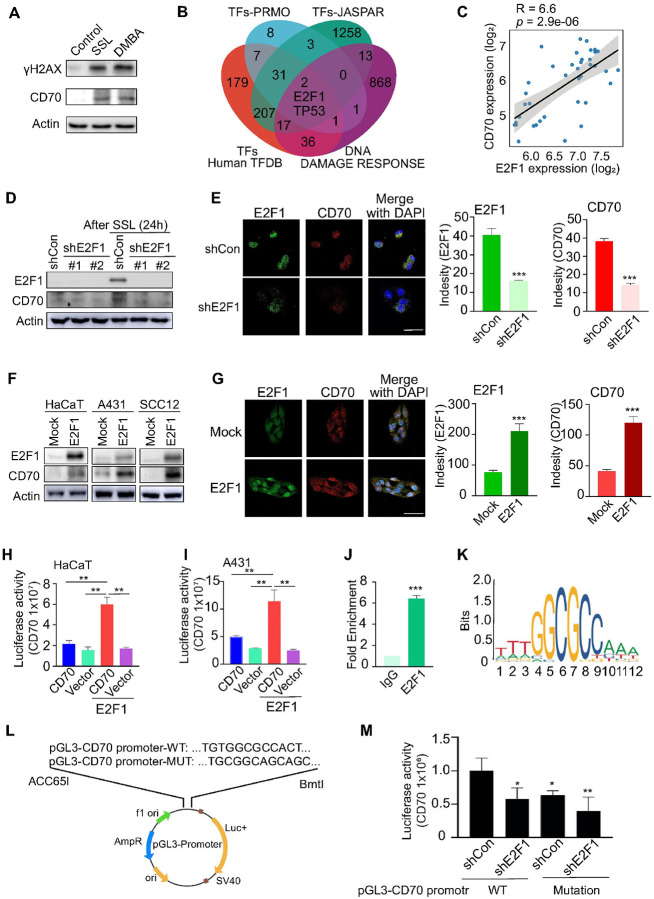
Solar UV–induced DNA damage response mediates CD70 expression *via* E2F1. **(A)** CD70 expression increases 24 h after SSL and DMBA treatment in HaCaT keratinocytes. **(B)** The potential of transcript factors from TFs-JASPAR, TFs-PRMO and TFs Humna TFDB overlapped with GOBP_DNA_DAMAGE_RESPONSE from GSEA MSigDB identity E2F1 and TP53 as potential transcript factors mediating CD70. **(C)** Correlation analysis of merged GEO datasets reveals a positive association between CD70 and E2F1 expression. **(D)** SSL exposure upregulates E2F1 and CD70 expression, whereas E2F1 knockdown attenuates CD70 induction in HaCaT cells, confirmed by Western blotting. **(E)** E2F1 knockdown attenuates CD70 expression in SSL-treated HaCaT cells as assessed by immunofluorescence (left). E2F1 is shown in green, CD70 is shown in red, and nuclei are counterstained with DAPI (blue). Scale bar: 50 μm. The intensity was quantified (right) using the ZEISS ZEN 3.7 software program. **(F)** E2F1 overexpression increases CD70 levels in HaCaT, A431, and SCC12 cells. **(G)** This result was validated by immunofluorescence in A431 cells. Luciferase assays show that E2F1 overexpression enhances CD70 promoter activity in **(H)** HaCaT and **(I)** A431 cells. The statistical analysis was determined by one-way ANOVA, Tukey’s HSD. **(J)** ChIP assay demonstrates E2F1 binding to the CD70 promoter region. **(K)** Predicted CD70 promoter motifs show E2F1 binding sites as assessed by JASPAR and MEME. **(L)** A CD70 promoter construct with mutated E2F1 binding sites was generated and sequence-verified by Genewiz. **(M)** Luciferase assays show that the mutated CD70 promoter reduces CD70 activity in HaCaT cells. Statistical significance was determined by one-way ANOVA followed by Tukey’s HSD test (H, I, M) and by Student’s *t*-test (E, G, J) (*p < 0.05; **p< 0.01; ***p < 0.001).

Solar UV irradiation induces persistent DNA damage, a key driver of skin carcinogenesis (3, 4). We found that solar simulated light (SSL) markedly increased CD70 expression in keratinocytes. Similarly, the chemical carcinogen 7,12-dimethylbenz[a]anthracene (DMBA), a classical DNA-damaging initiator, elevated CD70 levels (Fig. 3A). Other genotoxic agents, including camptothecin (CPT), etoposide (ETP), and aphidicolin (APH), also enhanced CD70 expression (**Supplementary Fig. 2A**), indicating that CD70 is a DNA damage–inducible gene and may function as a downstream effector of UV-driven genotoxic stress.

To identify transcription factors mediating CD70 induction, we queried JASPAR, PROMO, and HumanTFDB databases and overlapped predicted CD70-binding factors with the GOBP_DNA damage response gene set. E2F1 and TP53 emerged as top candidates (Fig. 3B). Meta-analysis of GSE2503 and GSE42677 datasets revealed a strong positive correlation between CD70 and E2F1 expression (R = 0.66, *p* = 2.9 × 10^−6^) (Fig. 3C), and a negative correlation with TP53 (R = − 0.49, *p* = 0.0014) (**Supplementary Fig. 2B**). E2F1 expression was also significantly elevated in AK and cSCC compared with normal skin (**Supplementary Fig. 2C**).

Functionally, E2F1 knockdown markedly reduced CD70 expression in A431 and SCC12 cells (**Supplementary Fig. 2D**) and inhibited cSCC cell proliferation (Supplementary Fig. 2E). SSL treatment increased both E2F1 and CD70 expression, whereas E2F1 silencing diminished CD70 levels, as confirmed by Western blot (Fig. 3D) and immunofluorescence (Fig. 3E, **Supplementary Fig. S3**). Conversely, E2F1 overexpression elevated CD70 in HaCaT, A431, and SCC12 cells (Fig. 3F), a result further verified by immunofluorescence showing robust CD70 induction (Fig. 3G).

Mechanistically, luciferase reporter assays demonstrated that E2F1 overexpression activated the CD70 promoter in HaCaT (Fig. 3H) and A431 (Fig. 3I) cells. ChIP-qPCR confirmed direct E2F1 binding to predicted motifs within the CD70 promoter (Fig. 3J). Motif analyses (JASPAR, MEME) identified a canonical E2F1 binding site (−879 to – 890 bp relative to the transcription start site; Fig. 3K), and site-directed mutagenesis (TGTGGCGCCACT → TGCGGCAGCAGC; Fig. 3L) significantly attenuated E2F1-driven promoter activity (Fig. 3M). Together, these results establish E2F1 as a direct transcriptional activator of CD70 downstream of UV-induced DNA damage, linking genotoxic stress to oncogenic and inflammatory signaling in skin epithelial cells.

### CD70 knockdown alters pro-tumorigenic signaling pathways

**Figure 4:**
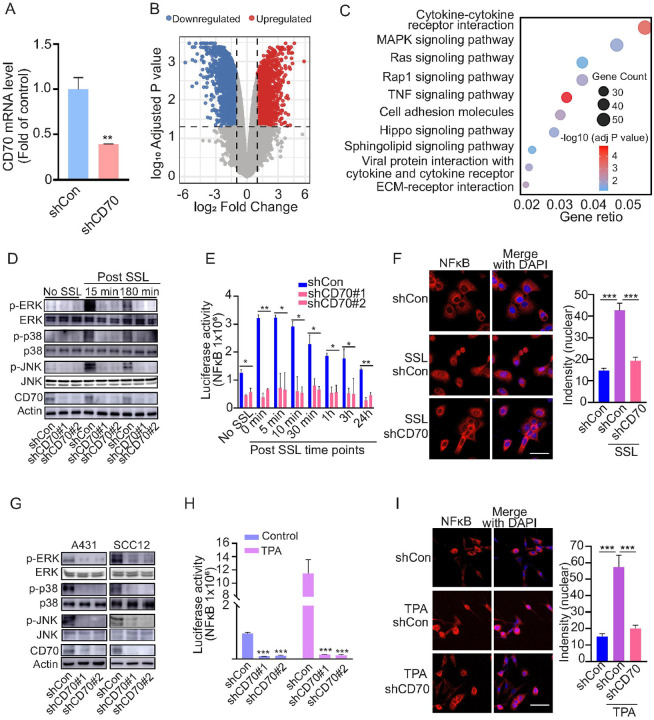
CD70 knockdown suppresses pro-tumorigenic signaling. **(A, B)** RNA-seq of SSL-exposed HaCaT cells with or without CD70 silencing reveals distinct gene expression changes. **(C)** KEGG analysis highlights cytokine–cytokine receptor interaction and MAPK signaling as the most altered pathways. **(D)** Knockdown of CD70 attenuates SSL-induced ERK, p38, JNK phosphorylation in HaCaT cells. Knockdown of CD70 attenuates NF-κB nuclear translocation as assessed by luciferase assay **(E)**and immunofluorescence **(F)** in HaCaT cells. **(G)**Knockdown of CD70 suppress ERK, p38, JNK phosphorylation in A431 and SCC12 cells. **(H)** Knockdown of CD70 suppresses NF-κB activation with or without TPA treatment in A431 cells. **(I)** Knockdown of CD70 decreases TPA induced NF-κB nuclear translocation in A431 cells (left). Immunofluorescence images: scale bar, 50 μm. Signal intensity was quantified (right) using ZEISS ZEN 3.7 software. Data are presented as means ± SE. Statistical significance was determined by one-way ANOVA followed by Tukey’s HSD test (*p < 0.05; **p < 0.01; ***p < 0.001).

To examine pathways regulated by CD70, we performed RNA-seq of HaCaT keratinocytes exposed to solar-simulated light (SSL) with or without CD70 knockdown. Differential expression analysis identified 1,275 upregulated and 2,333 downregulated genes (fold change ≥ 2, p < 0.05) Fig. 4A, B). KEGG enrichment highlighted cytokine–cytokine receptor interaction and MAPK signaling as the most affected pathways (Fig. 4C).

Heatmap visualization showed that CD70 knockdown led to reduced expression of pro-inflammatory mediators (e.g., IL1B, CCL20, CXCR2, CCL2, TNFRSF1A) and MAPK pathway components (e.g., IL1B, MAPK3, MAP2K6, MAPK13, JUND, FOS) (**Supplementary Fig. 4A, B**). These results indicate that CD70 regulates keratinocyte pro-inflammatory mediators and MAPK signaling, likely via NF-κB–mediated transcriptional control of cytokines and chemokines(26, 36–38). RT-PCR analysis confirmed that knockdown of CD70 decreased IL1B expression, a gene involved in both cytokine–cytokine receptor interactions and MAPK signaling, in HaCaT and A431 cells (**Supplementary Fig. 4C, D**). MAPK signaling (ERK, p38, JNK) is rapidly activated by UV and mediates keratinocyte stress responses, proliferation, and pathways driving SCC initiation and progression (39). Genetic inhibition of MAPK signaling suppresses UV-induced skin carcinogenesis in preclinical models (40, 41). SSL exposure induced phosphorylation of ERK, p38, and JNK and nuclear translocation of NF-κB p65 in HaCaT cells; CD70 knockdown markedly attenuated these responses, as shown by Western blotting (Fig. 4D), luciferase assays (Fig. 4E), and immunofluorescence (Fig. 4F, **Supplementary Fig. 5A**).

Similar results were observed in A431 and SCC12 cells, where CD70 knockdown reduced ERK, p38, and JNK phosphorylation (Fig. 4G) and NF-κB transcriptional activity (Fig. 4H). Immunofluorescence confirmed impaired NF-κB nuclear translocation (Fig. 4I; **Supplementary Fig. 5B**). Together, these data indicate that CD70 amplifies NF-κB and MAPK signaling in response to UV, promoting cSCC cell growth.

### CD70 expression in fibroblasts promotes tumor–stroma crosstalk and cSCC growth

**Figure 5:**
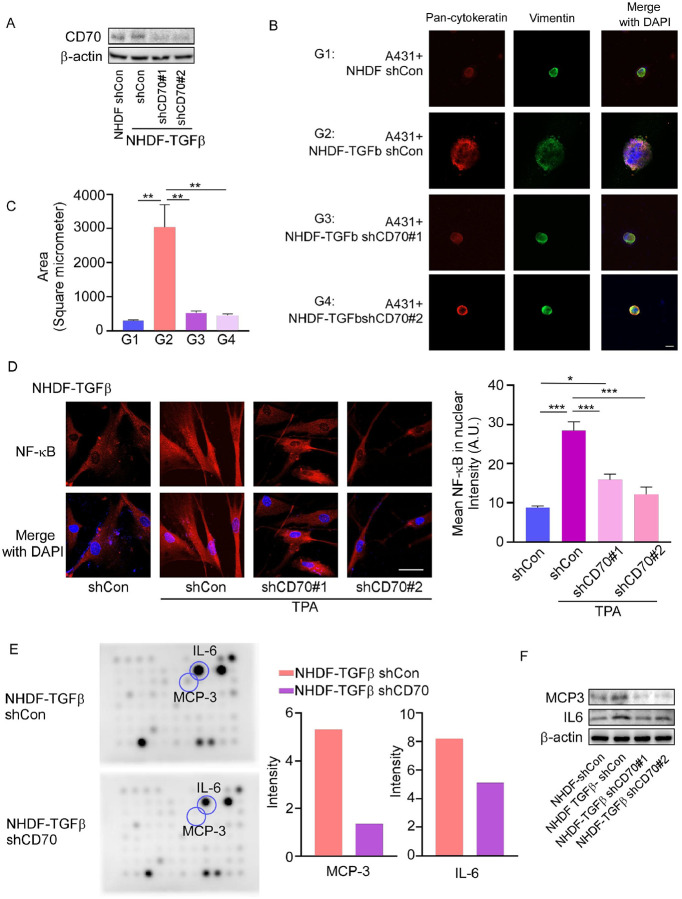
CD70 expression in fibroblasts promotes tumor–stroma crosstalk. **(A)** Western blot confirming knockdown of CD70 in NHDF1-TGFβ fibroblasts. **(B, C)** 3D co-culture assays showing that NHDF1-TGFβ fibroblasts enhance A431 spheroid growth, whereas knockdown of CD70 suppresses spheroid growth. **(D)** Immunofluorescence analysis showing that knockdown of CD70 reduces NF-κB nuclear translocation in NHDF1-TGFβ fibroblasts upon TPA stimulation (left). Scale bar: 50 μm. Nuclear intensity was quantified (right) using the ZEISS ZEN 3.7 program. **(E)** Cytokine array of conditioned media (CM) showing decreased IL6 and MCP3 secretion after knockdown of CD70 (left). Spot intensities were quantified using ImageJ, and cytokine expression levels are presented in graphical form (right). **(F)**Western blot validation showing reduced IL6 and MCP3 expression in fibroblast conditioned media (CM) after knockdown of CD70. Data are presented as mean values ± SE, with statistical significance determined by one-way ANOVA with Tukey’s HSD (*p < 0.05; **p < 0.01; ***p < 0.001).

Our previous results demonstrate that CD70 is not only expressed in epidermis but also detected in dermis (Fig. 1C, D) Among stromal elements, CAFs drive drive tumor progression primarily through secretion of pro-inflammatory cytokines and growth factors (26–29). To model CAF activation, normal human dermal fibroblasts (NHDF1) were chronically treated with TGF-β to generate NHDF1-TGFβ cells (26). Stable shCD70 and shCon NHDF1-TGFβ lines were established (Fig. 5A).

Conditioned media (CM) from NHDF1-TGFβ fibroblasts enhanced A431 and SCC12 cell growth compared with CM from normal NHDF1 cells, whereas CM from shCD70 NHDF1-TGFβ cells markedly suppressed growth (**Supplementary Fig. 6A, B**). In contrast, CM from shCD70 NHDF1-TGFβ cells significantly suppressed A431 and SCC12 growth relative to CM from shCon NHDF1-TGFβ cells (**Supplementary Fig. 6A, B, respectively**). In 3D spheroid co-cultures, A431 cells co-cultured with NHDF1-TGFβ fibroblasts formed more compact spheroids than with normal fibroblasts, and CD70 knockdown in NHDF1-TGFβ cells significantly reduced spheroid formation (Fig. 5B, C).

Mechanistically, CD70 knockdown in NHDF1-TGFβ fibroblasts suppressed TPA-induced NF-κB nuclear translocation (Fig. 5D) and and reduced secretion of IL6 and MCP3, as confirmed by Cytokine Array and Western blot (Fig. 5E, F; **Supplementary Fig. 7**). Notably, NF-κB is a well-established regulator of IL6 and MCP3 expression (42–45) and therefore its decreased activation and translocation induced by decreased CD70 correspond well. These results suggest that CD70 mediates fibroblast-derived cytokine production through the NF-κB pathway.

**Figure 6:**
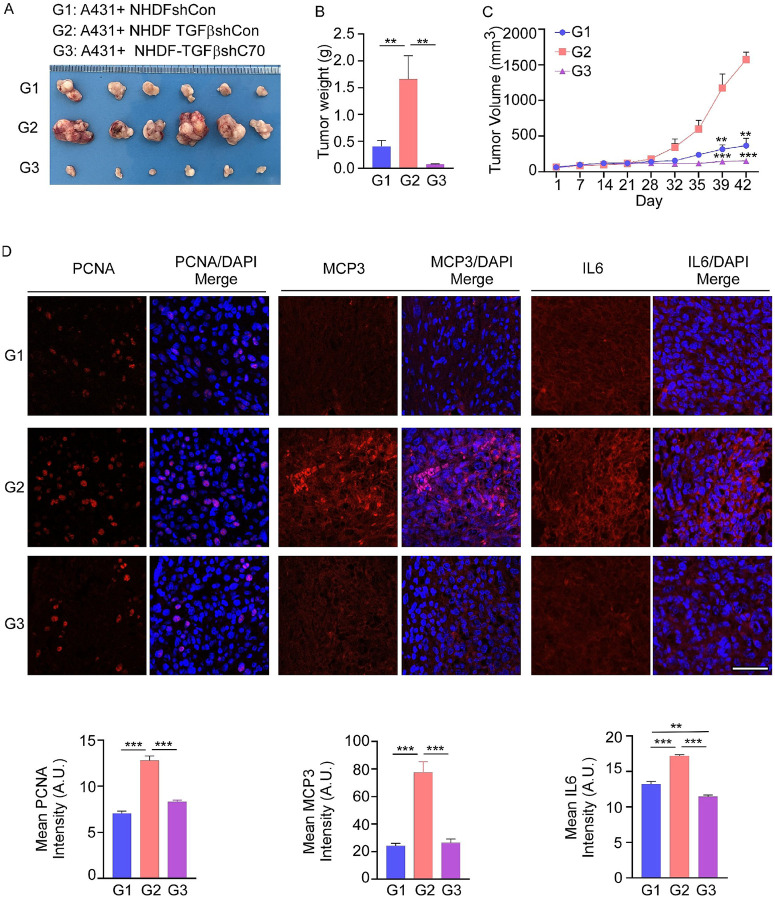
Fibroblast-derived CD70 enhances cSCC tumor growth *in vivo*. **(A)** Tumor growth curves of nude mice co-injected with A431 cells and fibroblasts show accelerated tumor growth with NHDF1-TGFβ fibroblasts compared with NHDF1 controls, and suppression with CD70-silenced NHDF1-TGFβ fibroblasts. Tumor **(B)** weights and **(C)** volumes at the endpoint of the study show reduced tumor burden in the CD70 knockdown group. **(D)**Immunofluorescence staining of xenograft tumors showing decreased PCNA, IL6, and MCP3 expression in tumors derived from CD70-silenced fibroblasts (upper). The scale bar: 50 μm. Intensity was evaluated (lower) using the ZEISS ZEN 3.7 software program. Data are presented as means ± SE, with statistical significance determined by one-way ANOVA with Tukey’s HSD (B, D) and Student’s *t* test (C) (**p < 0.01; ***p < 0.001).

*In vivo*, co-injection of A431 cells with fibroblasts into nude mice enhanced tumor growth. Compared with co-injection of A431 cells with shCon NHDF1 fibroblasts, co-injection with NHDF1-TGFβ shCon fibroblasts further accelerated tumor growth. In contrast, knockdown of CD70 in NHDF1-TGFβ fibroblasts significantly suppressed tumor size (**representative images**, Fig. 6A), weight (Fig. 6B), and volume (Fig. 6C) compared with the shCon group. Tumors from the CD70 knockdown group also exhibited reduced expression of PCNA and cytokines IL6, and MCP3 (Fig. 6D).

## Discussion

Our study identifies CD70 as a UV-inducible, DNA damage–responsive mediator in NMSC, linking genotoxic stress, inflammatory signaling, and crosstalk between cSCC cells and fibroblasts. CD70 is upregulated in sun-exposed skin, AK, and cSCC, indicating its potential as an early molecular marker of UV-driven carcinogenesis. This extends prior work connecting UV-induced DNA damage and inflammation to skin cancer (4) and positions CD70 as a direct effector linking these processes.

We demonstrate that UV-induced DNA damage induces CD70 in keratinocytes, revealing a previously unrecognized connection between environmental genotoxic stress and CD70 signaling. While CD70 is classically studied in immune activation through CD27 (10–13), its role as a stress-responsive molecule in epithelial cells has been largely unexplored. This induction is directly mediated by E2F1, a DNA damage–associated transcription factor (46, 47), which binds the CD70 promoter and activates transcription following UV or genotoxic treatment. E2F1 is a central mediator of DNA damage responses and oncogenic processes, regulating cell cycle progression, apoptosis, and DNA repair (48, 49). Our findings suggest that E2F1 promotes cSCC not only through cell growth but also via direct CD70 induction, consistent with reports of genotoxic stress–driven CD70 expression (22, 50) revealing a novel E2F1–CD70 axis connecting UV-induced DNA damage to oncogenic and inflammatory signaling.

Functionally, CD70 amplifies pro-tumorigenic signaling by engaging MAPK and NF-κB pathways, shaping a cytokine-rich microenvironment that promotes keratinocyte proliferation and cSCC growth. Consistent with our findings, CD70 clustering can initiate reverse signaling through its cytoplasmic tail, engaging PI3K/AKT, MAPK and NF-κB pathways that promote proliferation, EMT, invasion, and immune-evasive phenotypes in tumor models (10, 14–16) (51–53). Activation of MAPK and NF-κB converges on inflammatory mediators and tumor-promoting genes such as IL1B, which facilitate tumor initiation, fibroblast activation, angiogenesis, EMT, and immune suppression (54–58). Our RNA-seq and cytokine array analyses support CD70 as an upstream regulator of a feed-forward inflammatory module driving oncogenesis.

The role of CD70 in stromal signaling represents a previously unrecognized mechanism in skin carcinogenesis, although previous studies have shown that CAFs form a distinct stromal subset that promotes tumor cell migration, drives regulatory T cell accumulation, and correlates with poor survival (59–61). Our findings reveal that fibroblast-derived CD70 amplifies cytokine production via NF-κB, thereby enhancing keratinocyte proliferation and 3D tumor spheroid formation. *In vivo*, co-injection of CD70-expressing fibroblasts with cSCC cells accelerates tumor growth, whereas CD70 knockdown impairs tumor progression and reduces PCNA, IL6, and MCP3 expression, confirming its functional relevance in the tumor microenvironment.

Beyond its direct effects on epithelial proliferation, CD70 shapes the tumor immune microenvironment by modulating the cytokine and chemokine milieu. Inflammatory mediators such as IL1β, IL6, and MCP-3 (CCL7) not only drive SCC cell growth but also recruit and condition myeloid populations, including monocytes, tumor-associated macrophages, and MDSCs, thereby promoting a suppressive, tumor-permissive niche (62–66). CD70 in epithelial cells and fibroblasts may thus coordinate tumor progression by directly activating MAPK and NF-κB in tumor cells while indirectly sustaining immune evasion. Limitations include reliance on in vitro and murine models, incomplete demonstration of causal roles in vivo, and lack of human longitudinal or preclinical pharmacologic data, emphasizing the need for further validation.

CD70 functions as a dual-role node regulating epithelial proliferation, inflammatory signaling, and stromal crosstalk. Its rapid induction by UV-induced DNA damage, coupled with low basal expression in normal tissues, underscores its potential as a selective therapeutic target. Anti-CD70 therapies under investigation in hematologic malignancies and solid tumors (10–13). Our data suggest that CD70-targeted strategies could be repurposed for NMSC to disrupt both epithelial and stromal oncogenic programs. Future studies should investigate CD70/CD27 interactions across immune populations and assess CD70-targeted antibodies, antibody–drug conjugates, and small-molecule inhibitors for preventing or treating UV-induced cSCC.

**Figure 7:**
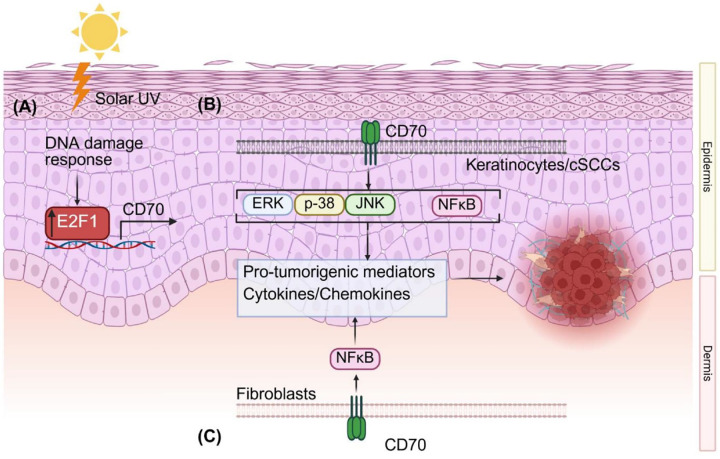
Schematic model of CD70-mediated signaling in solar UV–induced cSCC. Solar UV triggers **(A)** DNA damage responses in keratinocytes, leading to increased E2F1 expression and induction of CD70 expression. **(B)** CD70 promotes activation of MAPK (ERK, p38, JNK) and NF-κB signaling pathways, driving the production of pro-inflammatory and pro-tumorigenic mediators such as IL1B. **(C)** Fibroblast-derived CD70 further enhances NF-κB activation and cytokine secretion, including IL6 and MCP3, reinforcing tumor–stroma crosstalk. Together, these mechanisms link UV-induced genotoxic stress to CD70-driven oncogenic signaling and cSCC development.

In conclusion, we identify CD70 as a central integrator of DNA damage, inflammatory signaling, and tumor–stroma interactions in UV-driven NMSC. Solar UV–induced DNA damage robustly induces CD70 expression via E2F1, which in turn mediates keratinocyte and cSCC cell proliferation through MAPK and NF-κB signaling, driving the expression of inflammatory mediators and tumor-promoting factors such as IL1B. In addition, CD70 expressed in fibroblasts amplifies cytokine and chemokine production, including IL6 and MCP-3, which not only support cSCC growth, but also modulate the tumor microenvironment (Fig. 7). Together, our findings reveal a previously unrecognized mechanism linking environmental genotoxic stress to pro-tumorigenic signaling and provide a compelling rationale for developing CD70-targeted strategies for skin cancer prevention and therapy.

## Supplementary Material

Supplementary Files

This is a list of supplementary files associated with this preprint. Click to download.

- SupplementaryFigurelegendsCD70.docx
- SupplementaryMethodsCCD.docx
- SupplemetaryMaterialWB.pdf
- SupplementaryFigures.pdf

## Data Availability

The RNA-seq datasets generated during this study are available from the corresponding author upon reasonable request.
